# Phenotypic Analysis of BrdU Label-Retaining Cells during the Maturation of Conducting Airway Epithelium in a Porcine Lung

**DOI:** 10.1155/2019/7043890

**Published:** 2019-02-27

**Authors:** Yuanyuan Jia, Xuehong You, Ningxia Ma, Hui Li, Guopan Liu, Ying Wang, Jing Xue, Juan Shi, Jun Wei, Yong Li, Jiali Yang, Xiaoming Liu

**Affiliations:** ^1^Key Laboratory of Ministry of Education for Conservation and Utilization of Special Biological Resources in the Western, Ningxia University, Yinchuan, Ningxia 750021, China; ^2^College of Life Science, Ningxia University, Yinchuan, Ningxia 750021, China; ^3^College of Clinical Medicine, Ningxia Medical University, Yinchuan, Ningxia 750004, China; ^4^Institute of Human Stem Cell Research, General Hospital of Ningxia Medical University, Yinchuan, Ningxia 750004, China; ^5^Center of Laboratory Medicine, General Hospital of Ningxia Medical University, Yinchuan, Ningxia 750004, China; ^6^Department of Anatomy and Cell Biology, Carver College of Medicine, University of Iowa, Iowa City, Iowa 52242, USA

## Abstract

Stem/progenitor cells have recently been demonstrated to play key roles in the maturation, injury repair, and regeneration of distinct organs or tissues. Porcine has spurred an increased interest in biomedical research models and xenotransplantation, owing to most of its organs share similarities in physiology, cellular composition and size to humans. Therefore, characterization of stem/progenitor cells in porcine organs or tissues may provide a novel avenue to better understand the biology and function of stem cells in humans. In the present study, potential stem/progenitor cells in conducting airway epithelium of a porcine lung were characterized by morphometric analysis of bromodeoxyuridine (BrdU) label-retaining cells (LRCs) during the maturation of the lung. The results showed a pseudostratified mucociliary epithelium comprised of basal, ciliated, goblet, and columnar cells in the conducting airway of a porcine lung. In addition, the majority of primary epithelial cells able to proliferate in vitro expressed keratin 5, a subpopulation of these keratin 5-positive cells, also expressed CD117 (c-Kit) or CD49f (integrin alpha 6, ITGA6), implying that they might be potential epithelial stem/progenitor cells in conducting airway of a porcine lung. Lineage tracing analysis with a BrdU-labeled neonatal piglet showed that the proportion of BrdU-labeled cells in conducting airways decreased over the 90-day period of lung maturation. The BrdU-labeled epithelial cells also expressed keratin 14, mucin 5AC, or prosurfactant protein C (ProSP-C); among them, the keratin 14-positive cells were the most frequent BrdU-labeled epithelial cell type as determined by immunohistochemical and immunofluorescence staining. This study may provide valuable information on the biology and function of epithelial stem/progenitor cells in conducting airway of pigs and humans.

## 1. Introduction

The airway epithelium, a continuous pseudostratified population of cells lining the dichotomously branching airways, provides the barrier function that defends against inhaled gases, particulates, pathogens, and other xenobiotics [1–4]. In humans, the airway epithelium is comprised of 4 major cell types, including ciliated, secretory, column, and basal cells. While the ciliated, secretory, and columnar cells constitute the primary host defense barrier, basal cells are a subpopulation of proliferating cuboidal-shaped cells that provide the major stem/progenitor cell function from which other airway epithelial cells are derived [5–9]. Submucosal glands (SMGs) in the airway are beneath the epithelium and restricted to the highest reaches of the cartilaginous region of airway, which have been demonstrated as the stem cell niche of the cartilaginous tracheal airway [10, 11].

It has been well documented that there is a subset in the conducting airway epithelium are potential stem/progenitor cells responsible to the maintenance, remodeling, regeneration, and repair of the postnatal lung [7–10]. However, compared to murine lung stem cells, our understanding of adult human lung stem cells has just begun, partially owing to the relatively slow renewal of lung epithelial cells and the complex diversity of lung epithelial cell types. In this regard, the specific marker and biology of the lung stem cells remain largely unknown and need further identification. Additionally, in humans, like in other solid organs, the studies of human lung stem cells have been impeded by the limited source of tissues and ethnic concerns of in vivo study. Primary epithelial cells fail to replicate after a few passages and must be continuously harvested and isolated to complete each set of studies. In addition, molecular biology techniques to alter or delete the expression of genes of interest are difficult to achieve and sustain in primary epithelial cells [12]. Therefore, most of our current understanding of lung stem cell biology is using animal models, mainly the murine models. However, the murine lungs are very different from humans in terms of anatomy, epithelial cell composition and biology, lung physiology, and innate and acquired immune characteristics [13]. For example, club cells are present throughout the trachea to the bronchiolar epithelium and are the major type of secretory cells in the murine lung, while they are a rare cell type in human lungs, and goblet cells are the main secretory cells in human tracheobronchial airway [14]. Therefore, results of lung stem cell biology from murine models may less accurately reflect that of humans, as compared with those from animal models that are similar to humans in physiologically and size.

Encouragingly, pigs have been used as a model for biomedical research and a potential donor of organs for xenotransplantation in human, owing to the similarity between the pigs and human in characteristics of physiology, cell composition, and the structure and size of organs [11, 15, 16]. The anatomy, bronchoscopy such as bronchial tree, lobular division, and blood vessels, and the biomechanical and biochemical characterization of a porcine lung have been previously studied [17]. The similar size and anatomy of porcine and human organs make this model particularly beneficial for translational research in areas such as medical device development, therapeutics, and xenotransplantation [17]. Moreover, the porcine-to-nonhuman primate models offer an alternative that the availability of genomic, transcriptomic, and progressive proteomic tools for the analysis of this species [15]. In the respect of xenotransplantation, the porcine lung donors have been transplanted into baboons as a surrogate for a human recipient [18].

In view of the importance of a porcine model in biomedical researches and organ donors for xenotransplantation, as well as the necessity in understanding stem cell biology of the lung, the purpose of this study was the morphometric characterization of bromodeoxyuridine (BrdU) label-retaining cells (LRCs) in conduction airway epithelium during the maturation of porcine lungs.

## 2. Materials and Methods

### 2.1. Animals and Tissue Processing

**Figure 1:**
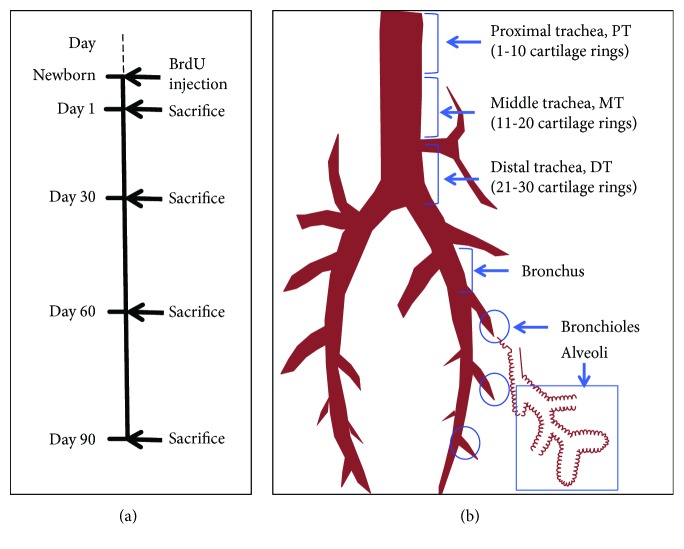
Timeline and tissues collected for analysis. (a) Timeline for postnatal maturation experiments of pig after the pulse of BrdU. (b) Illustration of the anatomic segments of conducting airway tissues analyzed in this study. PT, proximal trachea (1-10 cartilage rings); MT, proximal trachea (11-20 cartilage rings); DT, distal trachea (DT, 21-30 cartilage rings).

This study was approved by the ethics committee for the use of animals at Ningxia University. The genetic background of pigs used in this study was a three-crossbred pig (Duroc, Danish Landrace, and Yorkshire) [19]. Pigs were bred in the experimental farm of Ningxia University in Yinchuan. Six 3-month old adult pigs were employed for morphometric analysis. For the in vivo lineage tracing of label-retaining cells (LRC), twelve neonatal piglets had an intramuscular (i.m.) injection of 200 mg/kg of 5-bromo-2′-deoxyuridine (BrdU) dissolved in saline (Sigma, St. Louis, MO, USA); three of BrdU-labeled pigs were sacrificed, and the conducting airways (lung tissues) were collected at 1, 30, 60, and 90 days post BrdU pulse (Figure 1(a)). For the analysis, the conducting airway was segmented as the proximal trachea (PT, 1-10 cartilage rings), middle trachea (MT, 11-20 cartilage rings), distal trachea (DT, 21-30 cartilage rings), bronchus, and alveoli (distal lung) (Figure 1(b)) [17]. Collected tissues were fixed in 10% formalin neutral fixative or 4% paraformaldehyde fixative solution and embedded in paraffin or in the Tissue-Tek Optimal Cutting Temperature (OCT) compound (Sakura, Torrance, CA).

### 2.2. Isolation and Culture of Porcine Tracheal Epithelial Cells

The dissociation of porcine tracheal epithelial cells was performed per protocol previously described for humans [14]. Briefly, the distal trachea of pigs was essentially rinsed with PBS before it was incubated in dissociation solution containing pronase (1.5 mg/mL) and DNase I (10 *μ*g/mL) in DMEM with rotation at 4°C for 24 h. The enzymatic dissociation was terminated by adding FBS to a final concentration of 10%, and the epithelial cells were then collected by centrifugation at 500 × *g* for 10 min at 4°C. The cell pellet was resuspended in DMEM with 5% FBS, followed by seeding on tissue culture plates (Primera; Becton-Dickinson Labware, Franklin Lakes, NJ) for the adhesion of fibroblasts in 5.0% CO_2_ at 37°C for 2 h; then the nonadherent cells were collected by centrifugation and cultured in a Bronchial Epithelial Cell Growth Medium (BEGM) (Lonza, Basel, Switzerland). The fresh isolated cells were then for 24 h prior to be used for immunofluorescence analysis. For 3D sphere formation assay, above primary cells were resuspended in BEGM and then mixed 1 : 1 with growth factor-reduced Matrigel (BD Biosciences, Franklin Lakes, NJ) before they were cultured in 12-well Corning Transwell-clear permeable inserts (Corning, Corning, NY). The cells were incubated at 37°C, 5% CO_2_ atmosphere, and refreshed by adding BEGM to the lower chamber every other day for a week. The cultures were then used for accessing the spheres under a light microscope.

### 2.3. Histochemistry, Immunohistochemistry, and Immunofluorescence Assays

Hematoxylin and eosin (HE), Periodic acid-Schiff (PAS), and immunohistochemistry (IHC) staining were conducted on paraffin sections as described elsewhere [20, 21]. Paraffin sections were deparaffinized in xylene, followed by incubating in methanol containing 0.3% H_2_O_2_ for 30 min to inactivate endogenous peroxidase. Sections were then rehydrated in a series of graded ethanol according to histological standards. The antigen was retrieved by boiling in citrate buffer (pH 6.0) for 20 minutes, followed by cooling down to room temperature (RT). The sections were then blocked with blocking buffer (5% homologous serum with secondary antibody) at RT for 2 h, followed by incubating with corresponding primary antibodies (diluted in blocking buffer) in a humid chamber at 4°C overnight. Following washing three times for 5 min in PBS, a mixture of biotinylated secondary antibodies (Vector Laboratories, Burlingame, CA, USA) was applied at RT for 1 h. The antigen was detected with elite ABC kits and developed with a DAB Peroxidase substrate kit (Vector Laboratories, Burlingame, CA, USA). The antibodies and working dilutions used in this study are listed in Supplemental Table 1. Finally, the stained sections were briefly (10 sec) counterstained with hematoxylin (Gill's formula) (Vector Laboratories, Burlingame, CA, USA), followed by rinsing in running tap water for 5 min, dehydrating, clearing with xylene, and mounting in Permount (Fisher Scientific, Pittsburgh, PA, USA) [22]. To access the epithelial cell type of LRCs in the porcine conducting airways, paraffin sections with BrdU were colocalized with epithelial cell-specific marker by immunohistochemistry staining with anti-BrdU (Roche, Indianapolis, IN) and antiepithelial cell-specific antibodies (Supplementary Table 1). The stained sections were visualized under a light microscope, and the picture was captured using a Leica DFC300 F camera. Cover slides of primary epithelial cells or frozen sections of porcine lung tissues were performed by immunofluorescence staining. Immunofluorescence staining was conducted on 6 *μ*m frozen sections, using the appropriate primary and fluorescent-labeled secondary antibodies. Stained sections were mounted in a Vectashield Mounting Medium, which contained DAPI to demarcate the nucleus (H-1200, Vector Laboratories, Burlingame, CA).

### 2.4. Flow Cytometry Assay by Fluorescence-Activated Cell Sorting (FACS)

For FACS, the fresh isolated primary epithelial cells were cultured in BEGM for 48 h prior to be used for cytometry analysis staining. A million cells were suspended in 1 mL of 5% BSA in PBS and incubated with FITC mouse anti-Krt5 (FCMAB291F, Sigma, St. Louis, MO) and APC rat anti-CD49f (GoH3) (R&D Systems, Minneapolis, MN) or appropriate IgG isotype control at 4°C overnight, followed by washing before analysis. FACS analysis was performed on a flow cytometry system (Becton and Dickinson, San Jose, CA, USA) and analyzed using FlowJo software (Tree Star Inc., Ashland, OR, USA).

### 2.5. Quantification and Cell Counting

The composition of major epithelial cell types or BrdU-labeled cells in epithelium of a porcine lung was ascertained by following a standard histomorphometric analysis, recently set by the American Thoracic Society (ATS) [11]. Following the HE staining or immunohistochemistry staining on the serial transverse sections, epithelial cell counts were blindly made on every tenth section and then the cells with cell type-specific staining were counted by three senior technicians under a light microscope. The total number of epithelial cells on each section was ascertained by counting the hematoxylin-stained nuclei in the epithelial layer. The PAS staining was utilized as the standard for the identification of goblet cells in the lungs [23]. For each staining, at least three sections from each animal were evaluated. Representative images are shown in the corresponding figures. For each cell type, the percentage of BrdU-positive or specific epithelial cell type was represented as the percentage of positive stained cells in total counted epithelial cells. A total of six animals were analyzed for the composition of epithelial cells, and twelve animals were analyzed for BrdU label-retaining cells in this study.

### 2.6. Statistical Analysis

Statistical analysis was performed with the SPSS18.0 software. Quantitative data were evaluated by a one-way ANOVA and *t*-test for the comparison of differences between the two groups and presented as the mean ± standard deviation (SD). *p* values < 0.05 were accepted as a statistical difference, and *p* values < 0.01 were represented as a statistically significant difference.

## 3. Results

### 3.1. Morphometric Analysis of Epithelial Cells in the Conducting Airway of Adult Porcine

In order to morphometrically analyze epithelial cells in conducting airway of adult pigs, segments of the middle trachea (MT), bronchus, bronchioles, and distal lung (alveolar portion) were evaluated by HE and PAS histochemistry staining and IHC staining with antibodies against pulmonary epithelial cell-specific markers. HE staining showed that the porcine airway epithelium was located in the outermost layers of the airway lumen and composed of pseudostratified columnar epithelial cells (data not shown). Periodic acid-Schiff (PAS) staining is used for the identification of glycogen and the display of mucus. Periodic acid can make the intracellular polysaccharides oxidate into dialdehyde, combining with Schiff's colorless magenta and forming purple compounds, positioning in the cytoplasm. The PAS-positive populations are capable of secretory function and commonly considered to be the goblet cells [21, 24]. PAS staining revealed that a large amount of mucus secretion deposit in submucosal glands beneath the epithelium of cartilage airway and PAS-stained cells was also observed in the epithelium (data not shown), suggesting the existence of goblet cells in porcine airway epithelium that secrete mucus and provide a physical barrier to defend inhaled insults of gases, particulates, pathogens, and other xenobiotics.

**Figure 2:**
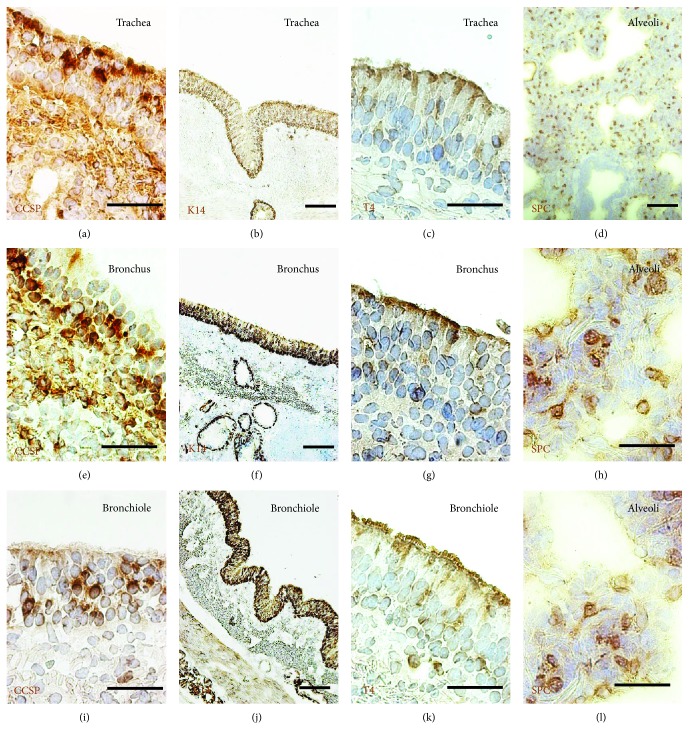
Composition of epithelial cell types in conducting airway epithelium of an adult porcine lung. Representative images of immunohistochemistry (IHC) staining with epithelial cell-specific markers were used for the identification of epithelial cell types of an adult porcine lung. (a, e, i) Immunohistochemistry staining of CCSP (brown) revealed that infrequent club cells are distributed in the region of the trachea, bronchus, and bronchiole. (b, f, j) Immunohistochemistry staining of K14 (brown) showed that basal cells are distributed in the region of the trachea, bronchus, and bronchiole. (c, g, k) Immunohistochemistry staining of T4 (brown) showed that ciliated cells are tapered along the tracheal-bronchial airway axis. (d, h, l) Immunohistochemistry staining of SPC (brown) showed the alveolar type 2 (AT2) cells in the alveoli region of a distal lung, counterstained with hematoxylin (blue). Bar = 50 or 100 *μ*m.

**Figure 3:**
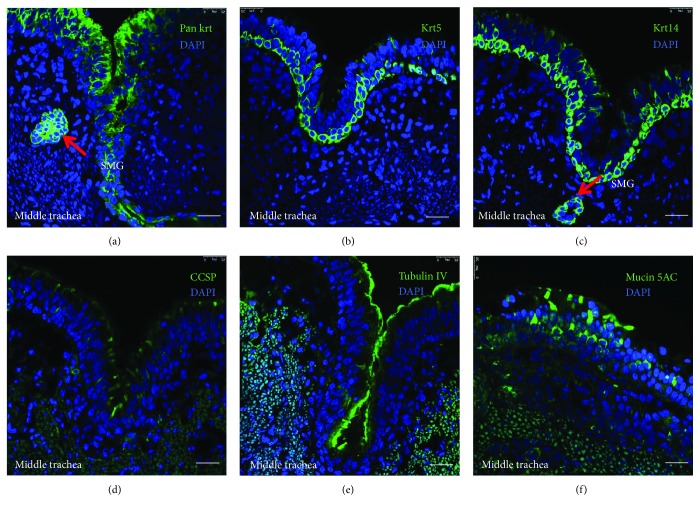
Immunofluorescence staining of epithelial cell-specific markers in the middle trachea of an adult porcine lung. Representative images of immunohistochemistry (IHC) staining with epithelial cell-specific markers were used for the identification of epithelial cell types in the middle trachea of an adult porcine lung. (a, b, c) Immunofluorescence staining for keratins of porcine tracheal epithelium, (a) pan keratin (green); (b) keratin 5 (Krt5) and (c) keratin 14 (Krt14) showed that basal cells were cuboidal-shaped and relatively short and contact the basement membrane and luminal cells. (d) Immunofluorescence staining for CCSP (green) revealed that infrequent cone-shaped club cells resided in the porcine tracheal epithelium. (e) Immunofluorescence staining for tubulin IV (green) showed the ciliated cells in the tip of epithelial layer. (f) Immunofluorescence staining for mucin 5AC (green) revealed that the secretory cells include morphologically and functionally distinct cells such as the goblet, serous, and club cells. Nuclear stained with DAPI (blue). Bar = 50 *μ*m.

IHC staining and IF staining were used to access the composition of major epithelial cell types in different conducting airway segments of adult porcine lungs, and the results show that Clara cell secretory protein- (CCSP-) positive club cells and keratin 14- (K14-) positive basal cells mainly distributed in the region of the trachea, bronchus, and bronchiole. However, CCSP-positive club cells were found infrequently in the large conducting airway. The distribution of tubulin IV- (T4-) positive ciliated cells tapered along the tracheal-bronchial airway axis. Prosurfactant protein C- (SPC-) positive alveolar type 2 (AT2) cells are mainly distributed in the distal alveolar region (Figure 2). The K14, CCSP, T4, and SPC have been demonstrated as specific epithelial cell markers of basal cells, club cells, ciliated cells, and AT2 cells, respectively [7, 9]. In this regard, basal cells are cuboidal-shaped and relatively short and contact the basement membrane and luminal cells; club cells are cone-shaped and rare in the porcine trachea; ciliated cells are column cells of porcine airway epithelium and capable of abundant cilia protruding; secretory cells include morphologically and functionally distinct cells, such as the goblet, serous, and club cells, and can be determined as PAS-stained cells or mucin-secreting cells. IF staining further confirmed findings in IHC staining, i.e., abundant mucin 5AC-positive secretory cells and rare CCSP-positive club cells in the epithelium of a porcine trachea (Figure 3).

Quantitative analysis of the composition of main epithelial cell types in different regions of airway (middle trachea, bronchus and bronchiole) revealed that the percentage of basal cell marker K14-, ciliated cell marker tubulin IV-, and club cell marker CCSP-expressing epithelial cells in the above examined airway of adult porcine lungs was comparable, although the composition of each epithelial cell type was varied (Table 1). Of interest, the mucin 5AC-expressing secretory cells were less frequent in bronchiole in comparison with that in the trachea and bronchus of an adult porcine lung (*p* < 0.05) (Table 1). However, the percentages of the main above distinct epithelial cell types tend to gradually decline along the airway axis from the proximal to distal conducting airway (Table 1). Of note, the distribution of basal cells tapered along the tracheal-bronchial airway axis and was absent in bronchioles of murine airways, but K14 basal cells were observed in the pseudostratified epithelium from the trachea down to small bronchioles of the distal lung in pigs, which was similar to that in humans.

### 3.2. *In Vivo* Lineage Tracing of BrdU-Labeled Epithelial Cells in the Conducting Airway during the Maturation of a Postnatal Porcine Lung

**Figure 4:**
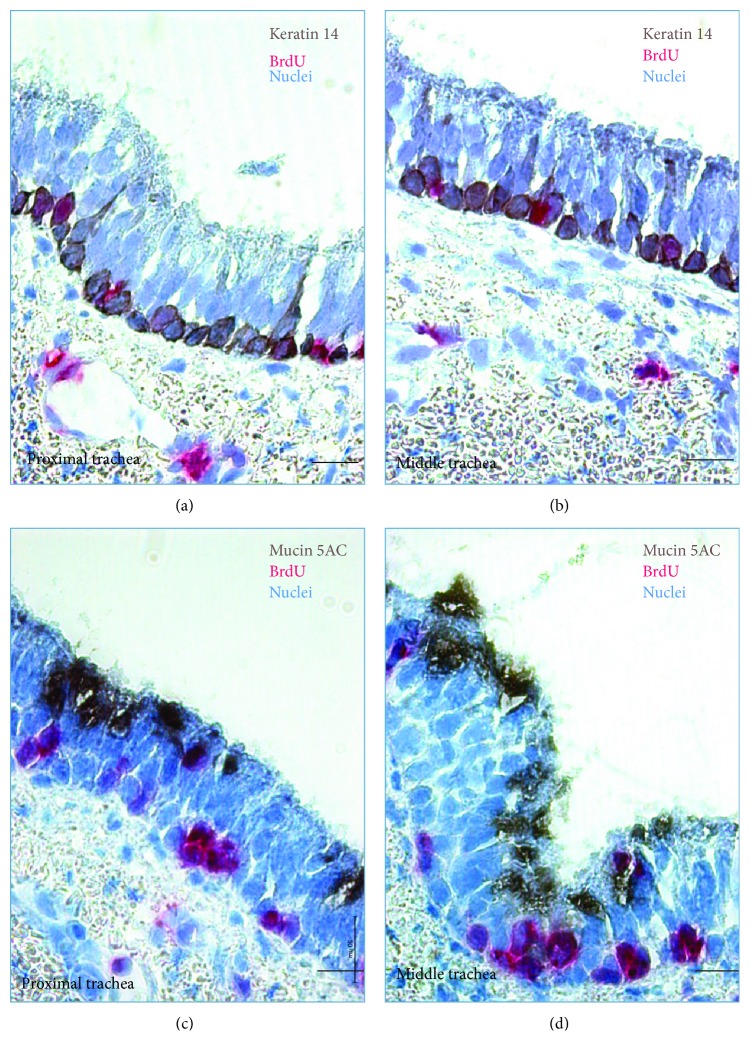
Immunohistochemistry staining of BrdU-labeled epithelial cells in porcine tracheal epithelium. Representative images of IHC staining of colocalization of BrdU and epithelial cell type marker keratin 14 and mucin 5AC in the trachea of porcine at day 1 post BruU pulse. (a, b) Colocalization of keratin 14 (brown) and BrdU (red) in the proximal (a) and middle (b) trachea of a pig lung determined by IHC. (c, d) Colocalization of mucin 5AC (brown) and BrdU (red) in the proximal (a) and middle (b) segments of the trachea in a pig lung determined by IHC and counterstained with hematoxylin (blue). Bar = 50 *μ*m.

**Figure 5:**
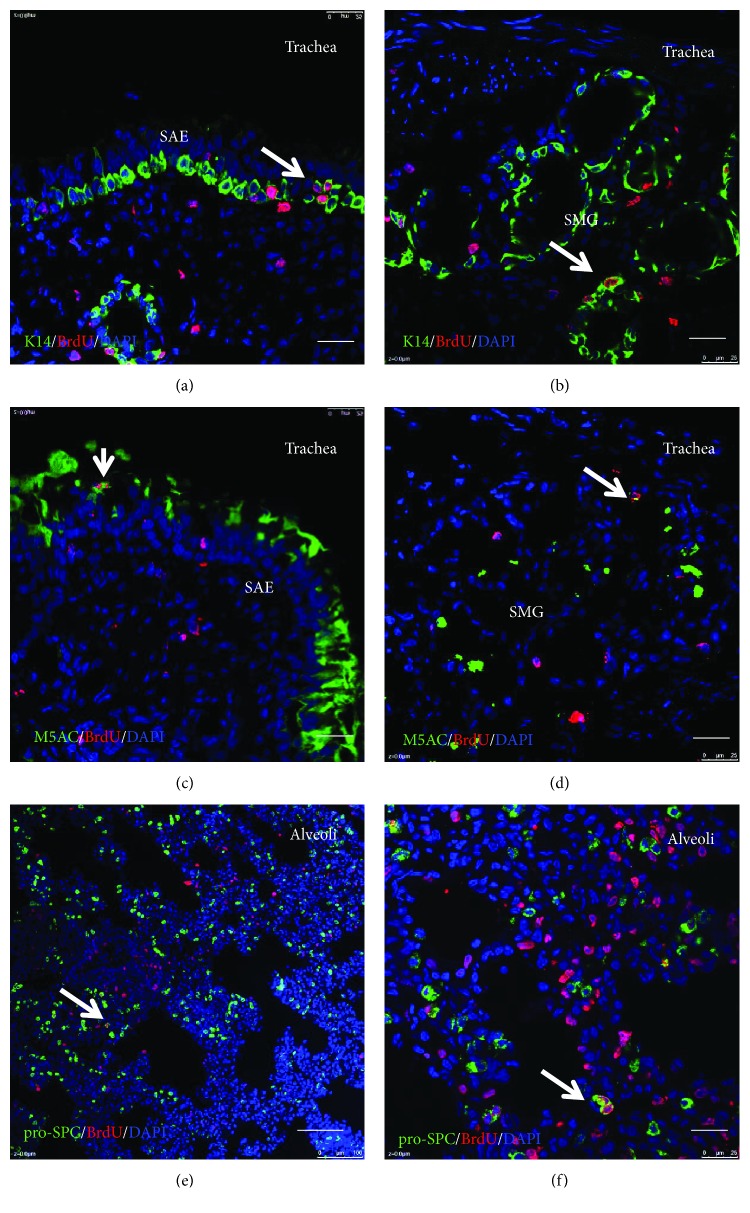
Efficiency of BrdU labeling in epithelial cells of a newborn porcine lung. The BrdU labeling efficiency was determined by immunofluorescence staining of BrdU and epithelial cell markers. Representative images of IF staining for colocalization of BrdU and epithelial cell type markers keratin 14, mucin 5AC, and ProSPC in the trachea and distal lung of porcine at day 1 post BruU pulse. (a, b) Colocalization of keratin 14 (green) and BrdU (red) in the surface airway epithelium (SAE) (a) and submucosal glands (SMG) (b) in the middle trachea of a porcine lung. (c, d) Colocalization of mucin 5AC (green) and BrdU (red) in the surface airway epithelium (SAE) (c) and submucosal glands (SMG) (d) in the middle trachea of a porcine lung. (e, f) Low (e) and high (f) magnifications of images of colocalization of ProSPC (green) and BrdU (red) in the alveolar region of a porcine distal lung. Nuclear stained with DAPI (blue). Bar = 50 *μ*m in (a, c, e). Bar = 100 *μ*m in (b, d, f).

Identification of regional stem/progenitor cell populations has been accomplished using a combination of techniques including *in vivo* lineage tracing of defined epithelial cell types, *in vitro* 3-dimensional culture assays to investigate the behavior of flow sorted epithelial cells, and ex vivo transplant assays following isolation of putative stem cells [8, 10, 25–28]. BrdU incorporation could indicate endoreduplication rather than cell proliferation and can be detected in a cell with antibodies. Therefore, lineage tracing BrdU-labeled cell in the airway epithelium of a newborn piglet was performed for up to 3 months. There was an increase in the length and diameter of the porcine conducting airway during postnatal maturation. The proportions of lineage-labeled K14-positive basal cells in the proximal trachea, middle trachea, distal trachea, bronchus, and bronchioles of a newborn pig were 24.93 ± 2.38%, 29.29 ± 1.90%, 22.63 ± 2.30%, 12.70 ± 1.80%, and 20.82 ± 5.61%, respectively, at day 1 post pulse of BrdU as determined by IHC colocalization, whereas the BrdU-labeled basal cells decreased over the tracing time period and even were rarely observed at postnatal 90 days (Figure 4 and Table 2). In consistent with that seen in K14-basal cells, mucin 5AC/BrdU double positive cells were observed over the course of the experiment, although its overall frequency was much less relative to that seen in K14-basal cells (Figure 4 and Table 3). IF staining further showed the colocalization of BrdU/keratin 14 and BrdU/mucin 5AC double positive cells in the surface airway epithelia (SAE) and submucosal glands (SMG) of a porcine trachea. In addition, BrdU/ProSP-C double stained AT2 cells were also observed in the distal lung of BrdU label porcine lungs as determined by the IF assay (Figure 5).

**Figure 6:**
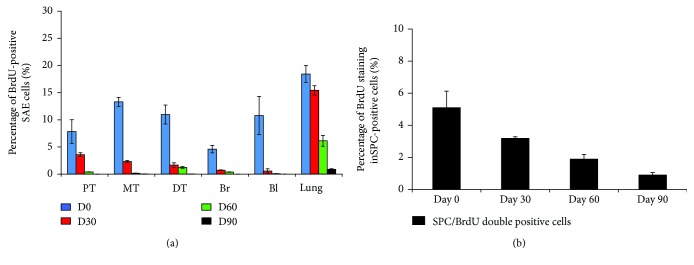
The overall BrdU-labeled epithelial cells during porcine lung maturation. The percentage of BrdU cells in the lung over 90 days was accessed by IHC assay with antibody to BrdU. (a) The percentage of BrdU-positive cells in SAE in conducting airway over postnatal days 1, 30, 60, and 90. (b) The percentage of BrdU-positive AT2 cells in the alveolar region of a distal lung at indicated time points was determined by IHC colocalization assay of BrdU/pro-SPC double staining. BI, bronchiole; Br, bronchus; DT, distal trachea; MT, middle trachea; PT, proximal trachea.

**Figure 7 fig7:**
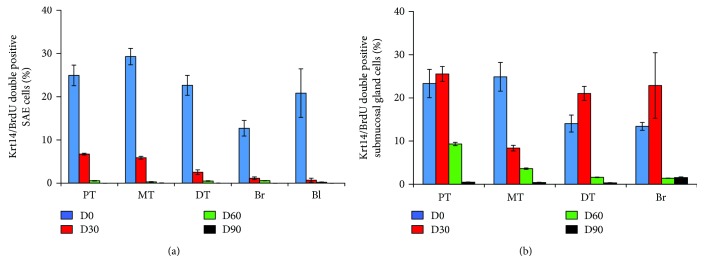
The percentage of BrdU-labeled keratin 14 basal epithelial cells in conducting airway during porcine lung maturation. The percentage of BrdU-labeled keratin 14-positive cells in the lung over 90 days was accessed by IHC colocalization assay with antibodies to BrdU and keratin 14. (a) The percentage of BrdU/keratin 14 double positive cells in total cells of SAE in indicated regions of conducting airway over postnatal days 1, 30, 60, and 90. (b). (a) The percentage of BrdU/keratin 14 double positive cells in total cells of SMGs beneath the SAE in indicated regions of conducting airway over postnatal days 1, 30, 60, and 90. BI, bronchiole; Br, bronchus; DT, distal trachea; MT, middle trachea; PT, proximal trachea.

**Figure 8:**
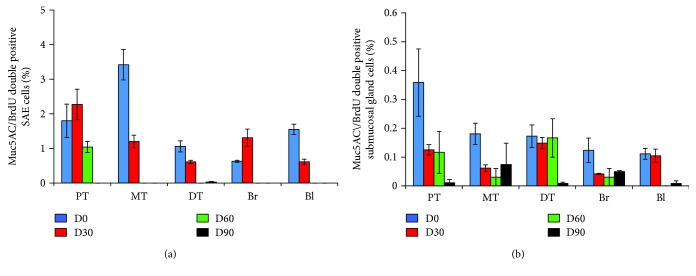
The percentage of BrdU-labeled mucin 5AC epithelial cells in conducting airway during porcine lung maturation. The percentage of BrdU-labeled mucin 5AC-positive cells in the lung over 90 days was accessed by IHC colocalization assay with antibodies to BrdU and mucin 5AC. (a) The percentage of BrdU/mucin 5AC double positive cells in total cells of SAE in indicated regions of conducting airway over postnatal days 1, 30, 60, and 90. (b). (a) The percentage of BrdU/mucin 5AC double positive cells in total cells of SMGs beneath the SAE in indicated regions of conducting airway over postnatal days 1, 30, 60, and 90. BI, bronchiole; Br, bronchus; DT, distal trachea; MT, middle trachea; PT, proximal trachea.

Quantitative analysis of the portion of BrdU-labeled epithelial cells in surface airway epithelium (SAE), submucosal glands (SMG), and alveolar region of conducting airway in a porcine lung pulsed with BrdU demonstrated that less than 15% epithelial cells in the SAE and SMGs were labeled with BrdU, although the overall BrdU-positive cells in the lung tissues were over 20% positive on the next day of pulse (Figure 6(a)). Of note, only about 5% of SPC-AT2 cells in the alveolar region of a distal lung were BrdU positive on the next day of pulse (Figure 6(b)). Over 25% of K14-basal cells in SAE (Figure 7(a)) and SMGs (Figure 7(b)) of cartilage airway could be labeled with BrdU on the next day of pulse. In addition, the proportions of BrdU-labeled mucin 5AC secretory cells were less than 5% and 0.4% of total cells in SAE (Figure 8(a)) and SMGs (Figure 8(b)) of conducting airway, respectively. It is noteworthy that the percentages of above BrdU-labeled cells were gradually declined over time and were less than 0.1% at the 90 days post BrdU pulse (Figures 6 [Fig fig7]–8).

### 3.3. The Expression of Potential Surface Markers of Epithelial Stem/Progenitor Cells in Porcine Bronchus

**Figure 9:**
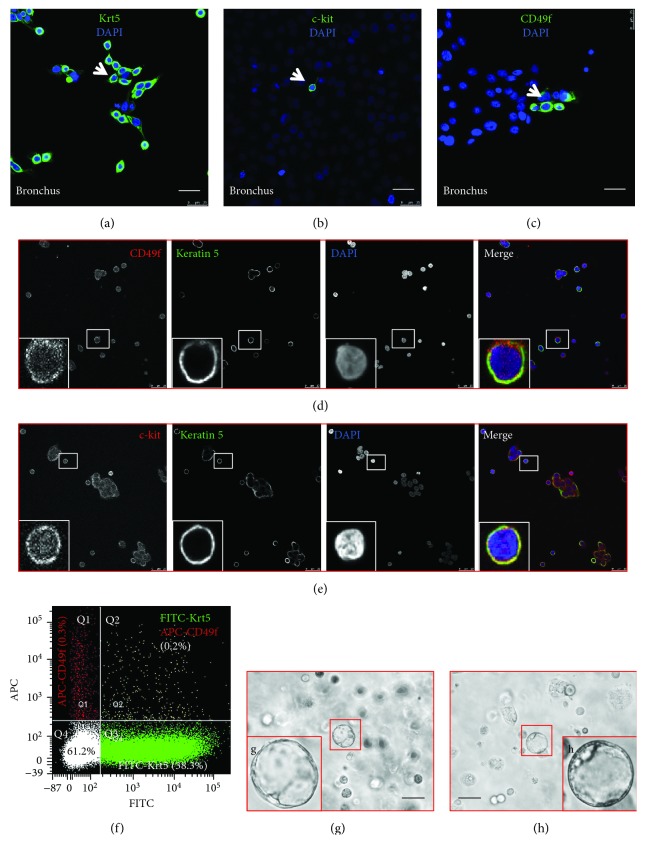
The expression of putative epithelial stem/progenitor cell markers and sphere formation assay of primary porcine bronchial epithelial cells. Freshly isolated primary porcine bronchial epithelial cells were cultured on cover slides in BEGM medium for 24 h prior to be employed for IF assay using indicated primary antibodies. (a) Immunofluorescence staining for keratin 5 (Krt5) (green) revealed that the majority of primary porcine bronchial epithelial cells expressed Krt5. (b) Immunofluorescence staining for c-Kit (CD117) (green) suggested that a subset of primary porcine bronchial epithelial cells expressed c-Kit. (c) Immunofluorescence staining for CD49f (ITGA) (green) showed that a subpopulation of primary porcine bronchial epithelial cells expressed CD49f. Nuclear stained with DAPI (blue). (d, e) Colocalization of Krt5 and c-Kit or CD49f in primary porcine bronchial epithelial cells. The colocation of putative lung epithelial stem/progenitor cell markers in primary porcine bronchial epithelial cells was ascertained by IF assay using indicated antibodies. Results showed that the coexpression of keratin 5 (green) and CD49f (red) (d) or c-Kit (red) (e) in a subset of keratin 5-positive cells. Nuclear stained with DAPI (blue). (f–h) Flow cytometry analysis of primary porcine bronchial epithelial cells cultured on a collagen precoated 100 mm dish in BEGM medium for 48 h and stained with fluorescence dye-labeled respective antibodies to Krt5 and CD49f or cultured in Matrigel for a 3D sphere formation assay. (f) A representative flow cytometry quadrant plot of primary porcine bronchial epithelial cells stained with FITC anti-Krt5 and APC anti-CD49f antibodies showed the portions of CD49f-positive (Q1, 0.3%), Krt5/CD49f double positive (Q2, 0.2%), Krt5-positive (Q3, 38.3%), and Krt5-negative (Q4, 61.2%) cells. (g, h) Representative images of the 3D culture of sphere formation assay showed spheres of primary porcine bronchial epithelial cells cultured for 7 days in Matrigel. G and H were enlarged insets in the corresponding selected areas of (b) and (c). Bars in (a–e) = 50 *μ*m in all images; bars in (g–h) = 100 *μ*m.

Next, we attempted to identify putative cell markers of porcine airway epithelial stem/progenitor cells. The expression of known putative lung epithelial stem cell markers was examined in primary epithelial cell cultures of a porcine bronchus by IF assay, in order to define whether stem cell-related transcriptional factors or markers were expressed in the epithelial cells in adult porcine lungs. The expression of CD117 (c-Kit), CD49f (integrin alpha 6 (ITGA6)), cyclin D1, Oct3/4, Sox 2, stem cell antigen-1 (Sca-1), stage-specific embryonic antigen-1 (SSEA-1), and thyroid transcription factor 1 (TTF-1) was examined in a native adult porcine bronchus by IF assay using antibodies listed in Supplementary Table 1. However, no expression of above antigens was observed with an exception of Sox2 and TTF-1 (data not shown). Interestingly, results of IF assay on primary bronchial epithelial cells showed that the majority of epithelial cells expressed keratin 5 (Krt5) (Figure 9(a)) and a subset of cells expressed c-Kit (CD117) (Figure 9(b)) and CD49f (ITGA6) (Figure 9(c)), despite the expression of other examined markers not detected (Figure 9). Furthermore, double staining of Krt5 and c-Kit (Figure 9(d)) or CD49f (Figure 9(e)) demonstrated the colocalization of Krt5 and c-Kit or CD49f in subpopulations of epithelial cells (Figure 9), and more abundant CD49f-positive cells relative to c-Kit-positive cells were observed in the primary culture of porcine bronchial epithelial cells (Figures 9(b) and 9(c)). Therefore, the abundance of Krt5-positive and/or CD49f-positive cells in the primary cultures of porcine bronchial epithelial cells was further analyzed by flow cytometry, and the percentages of Krt5-positive, CD49f-postive, and Krt5/CD49f double positive cells were 38.3%, 0.3%, and 0.2%, respectively (Figure 9(f)). Sphere formation assay also showed the potential of bronchial epithelial cells for differentiation and proliferation in vitro (Figures 9(g) and 9(h)). Together with other findings, this study suggests the existence of subsets of epithelial cells, such as CD49f- (ITGA6-) positive cells may be putative stem/progenitor cells in porcine conducting airway, which need to be further defined in the future.

## 4. Discussion

The diversity in mature epithelial cell types and their functions along the airway axis underscore the need for region-specific mechanisms to maintain the homeostasis and regenerate epithelium following an injury [22]. The conducting airway of the lung is lined with epithelial cells including basal cells, differentiated ciliated cells, and nonciliated secretory cells [22]. The nonciliated secretory cells include morphologically and functionally distinct cells such as the goblet, serous, and club cells [24]. The alveoli of terminal conducting airway are places of gas exchange, which are composed of alveolar type 1 (AT1) and type 2 (AT2) epithelial cells [29]. Several subsets of varied epithelial cell types have been identified as putative region-specific stem cells along the conducting airway axis [7, 9, 11, 30]. In this regard, a subset of basal cells in the conducting airway has been demonstrated to be multipotent stem cells responsible to tissue regeneration in adult lungs.

In the present study, the cellular composition of epithelial cells in the conducting airway of adult pigs and the putative airway epithelial stem/progenitor cells in maturing porcine lung was analyzed by accessing BrdU-labeled epithelial cells using IHC and IF assays and 3D sphere formation assay. Our data revealed a phenotypic similarity of porcine conducting airway epithelium to humans, which was comprised of basal, ciliated, goblet, and columnar cells. Lineage tracing assay showed that the proportion of BrdU-labeled cells in conducting airway waned over the 90-day period during the maturation of a porcine lung. The majority of BrdU-labeled epithelial cells also expressed keratin 14, mucin 5AC or ProSP-C, and the keratin 14-positive basal cells were the most frequent BrdU-labeled epithelial cell type in the cartilage conducting airway of a porcine lung. *In vitro* analysis of primary bronchial epithelial cells showed an expression of Krt5 in the majority of proliferative cells, and a subpopulation of these keratin 5-positive cells coexpressed CD117 (c-Kit) or CD49f (ITGA6), suggesting CD117 (c-Kit) and CD49f (ITGA6) might be potential porcine lung epithelial stem cell markers as seen in mouse and humans [8]. However, their potency as epithelial stem cells needs to be further investigated.

There is an increased line of evidences suggesting the existence of region-specific epithelial stem cells in the lung [8–10, 30]. However, we failed to identify the expression of stem cell-related gene in native adult pigs conducting airway by IHC and IF assays using available antibodies listed in Supplementary Table 1, with an exception of Sox2 and TTF-1 expression that was observed in respective alveolar epithelial cells and SMGs. There are two possible explanations for this result. First, the porcine airway epithelial stem/progenitor cell (EpiSPC) is dormant during a steady state and too rare to detect. Alternatively, the specificity of antibodies to identify the porcine airway epithelial stem/progenitor cell (EpiSPC) in our assay is almost generated with antigens from the mouse, rabbit, or human, which may have few cross immunogenicity to porcine airway epithelium. However, the identification of region-specific stem/progenitor cell populations has been accomplished using a combination of techniques by accessing the proliferation and differentiation behaviors and capacities of stem cells using *in vitro* 3-dimensional culture assays and ex vivo transplant assays following isolation of putative stem cell populations and *in vivo* lineage tracing assays to define epithelial cell types during the development, maturation, and injury repair of the lung [8, 10, 25–28]. However, most studies of stem cell identification are conducted in murine models due to the availability of animal models and less ethnic concerns. It will be a significant challenge in the study of stem cell identification in humans or even in nonhuman primates, since the availability of limited tissues and huge ethnic issues.

Pig is a large valuable animal model in biomedical research and an ideal donor of organs, including the lung for xenotransplantation, owing to the similarity in physiology and size of organs between pigs and humans. Therefore, the identification of stem cells in a porcine model may offer many advantages over murine models *in vitro* and *in vivo*. BrdU has been used for stem cell lineage tracing assay in mice for decades and more recently in monkeys [31]. Because of potential toxicity and ethnic concerns, it has been criticized of BrdU utilization in human, even for a short-term period [32–34]. In the present study, we attempted to interrogate the putative airway epithelial stem/progenitor cells by tracing BrdU-labeled epithelial cells during the maturation of a porcine lung. Our results were qualitatively consistent with previous findings where BrdU positivity was used as a proliferative marker in the mouse trachea and lung [11].

Of interest, the numbers of K14-positive basal cells and CCSP-positive club cells were consistent along the airway axis from the proximal to distal. This was different from the human lung where the numbers of basal cells and club cells were, respectively, decreased and increased with increased branching. Surprisingly, BrdU-labeled Muc5AC cells were also found in porcine conducting airway epithelial cells, but we could not rule out whether they were labeled during an early time point or differentiated from other BrdU-labeled progenitors, such as basal cells [8].

In addition, much higher ciliated cells were observed in small airways of a porcine lung relative to humans. This finding is worthwhile for further investigation. Equally noteworthy, the BrdU-labeled cells were observed in basal cells, secretory cells, and AT2 cells, but not in the ciliated cells. The failure of BrdU labeling of ciliated cells is indeed a terminally differentiated population at homeostasis, which is consistent with previously published results and confirms what they have observed using this lineage-labeling technique during airway development in mice [35], although a subset of ciliated cells has been demonstrated as epithelial stem/progenitor cells in a mouse trachea [35]. In addition, the overall number of ciliated cells in the airways remained constant throughout these experiments. Therefore, the ciliated cells must be replenished by the differentiation of stem cell populations, such as the basal cells in the trachea [6, 36]. Nonetheless, our study also revealed that the majority of BrdU-labeled cells throughout the conducting airway axis over maturation time of a porcine lung and the primary bronchial epithelial cells displayed a potency of proliferation differentiation as determined by a sphere formation assay. This is in line with the findings in murine models [6, 36].

In addition, CD117 (c-Kit) have been suggested as an epithelial stem cell marker in both human and murine lungs; a putative CD117 (c-Kit) positive population of human stem cells was able to generate both epithelial and endothelial cells in culture and promote airway and pulmonary vessel repair following cryoinjury in mice [37]. Several lines of evidence demonstrated that a subpopulation of epithelial cell expressing CD49f (ITGA6), CD104, and CD24 (low) has been identified as multipotent adult airway progenitor cells by in vitro Matrigel-based clonal assays. The subset of cells also exhibited an ability to generate airway epithelial cell as well as alveolar cell lineages [38, 39]. In this present study, we also observed the expression of Krt5/c-Kit and Krt5/CD49f subpopulations of primary porcine bronchial epithelial cells *in vitro*; in particular, the fraction of Krt5/CD49f subpopulations was as high as 0.2% of total primary bronchial epithelial cells in a porcine lung. However, their stem cell properties need to be further identification.

## 5. Conclusion

In conclusion, the phenotype and potential stem/progenitor cells in conducting airway epithelium during the maturation of porcine lung were analyzed. The results demonstrated a phenotypic similarity of conducting airway epithelium between pigs and other species, including humans and mice. *In vitro* sphere formation assay and *in vivo* BrdU-labeled cell tracing assay revealed that the basal cells, secretory cells, and AT2 cells might be epithelial stem/progenitor cell types responsible to epithelial cell proliferation during porcine lung maturation. *In vitro* culture of primary porcine bronchial epithelial cells further suggested that a subpopulation of epithelial cells coexpressing Krt5 and c-Kit or CD49f might be putative epithelial stem/progenitor cells in large conducting airway of a porcine lung. Our data thus offer useful information for studies in the biology and function of epithelial stem/progenitor cells in conducting airway of pigs and other species, including humans.

## Figures and Tables

**Table 1:** Composition of epithelial cell types in different segments of large conducting airway of an adult pig lung (%).

Segment/marker	Keratin 14	Tubulin IV	Mucin 5AC	CCSP
Trachea	37.3 ± 1.9	38.3 ± 3.4	19.8 ± 6.6	17.1 ± 3.9
Bronchus	32.1 ± 1.3	32.9 ± 2.2	14.3 ± 3.3	17.0 ± 4.2
Bronchiole	28.5 ± 1.3	25.4 ± 3.8	9.8 ± 1.3^**✝**^	19.7 ± 7.3

^**✝**^Compared to the trachea and bronchus, *p* < 0.05.

**Table 2:** Mean percentage of lineage-labeled keratin 14-positive cells ± SEM in different regions of porcine conducting airway over a time period of 90 days (%).

Time	Proximal trachea	Middle trachea	Distal trachea	Bronchus	Bronchioles
1 day	24.93 ± 2.38	29.29 ± 1.90	22.63 ± 2.30	12.70 ± 1.80	20.82 ± 5.61
30 days	6.71 ± 0.20	5.86 ± 0.34	2.54 ± 0.54	1.12 ± 0.33	0.68 ± 0.46
60 days	0.54 ± 0.06	0.28 ± 0.08	0.48 ± 0.07	0.56 ± 0.02	0.18 ± 0.10
90 days	0.00 ± 0.00	0.04 ± 0.04	0.01 ± 0.01	0.00 ± 0.00	0.00 ± 0.00

**Table 3:** Mean percentage of lineage-labeled Mucin 5AC-positive cells ± SEM in different regions of porcine conducting airway over a time period of 90 days (%).

Time	Proximal trachea	Middle trachea	Distal trachea	Bronchus	Bronchiole
0 day	1.80 ± 0.48	3.42 ± 0.44	1.06 ± 0.16	0.63 ± 0.03	1.55 ± 0.15
30 days	2.27 ± 0.44	1.20 ± 0.18	0.61 ± 0.05	1.32 ± 0.25	0.61 ± 0.08
60 days	1.04 ± 0.16	0.00 ± 0.00	0.00 ± 0.00	0.00 ± 0.00	0.00 ± 0.00
90 days	0.00 ± 0.00	0.00 ± 0.00	0.03 ± 0.02	0.00 ± 0.00	0.00 ± 0.00

## Data Availability

The data used to support the findings of this study are available from the corresponding author upon request.

## Supplementary Materials

Supplementary Materials Supplementary table 1: primary antibodies tested in this study. The detailed information on the antigen, host, catalog number, vendor, and applications of primary antibodies tested in this study was listed. ND: nonpositive signal was detected in this study. CCSP, Clara cell secretory protein; ICC, immunocytochemistry; IF, immunofluorescence; IHC, immunohistochemistry; ITGA6, integrin alpha 6; Sca-1, stem cell antigen-1; SSEA-1, stage-specific embryonic antigen-1; TTF-1, thyroid transcription factor 1.
