# Supplementary material for: Phenotypic Analysis of BrdU Label-Retaining Cells during the Maturation of Conducting Airway Epithelium in a Porcine Lung
Source: Stem Cells Int. 2019 Feb 27;2019:7043890. doi: 10.1155/2019/7043890 (PMC6415319; doi:10.1155/2019/7043890)
Supplement: Supplementary Materials — Supplementary table 1: primary antibodies tested in this study. The detailed information on the antigen, host, catalog number, vendor, and applications of primary antibodies tested in this study was listed. ND: nonpositive signal was detected in this study. CCSP, Clara cell secretory protein; ICC, immunocytochemistry; IF, immunofluorescence; IHC, immunohistochemistry; ITGA6, integrin alpha 6; Sca-1, stem cell antigen-1; SSEA-1, stage-specific embryonic antigen-1; TTF-1, thyroid transcription factor 1. [file 7043890.f1.pdf]

Supplementary table 1. Primary antibodies tested in this study*

Antigen	Host	Vendor	Catalog No.	Application
CCSP	Rabbit	Millipore	07-623	IF
CCSP	Rabbit	US Biologicals	C5828-03	IF, ICC, IHC
CD49f (ITGA6)	Rabbit	Biologend	313609	IF, ICC
CD49f (ITGA6)	Mouse	Millipore	MAB1961	ND*
CD104	Rabbit	Biologend	1223602	ND*
CD117 (c-Kit)	Rabbit	DAKO	A4502	ICC
CD117 (c-Kit)	Rabbit	Sigma	SAB4501648	ND*
CD133	Rabbit	Abcam	ab19898	ND*
Cyclin D1	Rabbit	Abcam	ab16663	ICC
Keratin 5	Rabbit	COVANCE	PRB-160	IF
Keratin 14	Mouse	Thermo	LL002	IF, ICC
Keratin 14	Rabbit	Lab vision	RB-9020	IF, ICC, IHC
Keratin 18	Mouse	Thermo	MS-142	IF
Mucin 5AC	Rabbit	Santa Cruz	sc-20118	IF, ICC, IHC
Oct3/4	Rat	R&D	MAB1759	ND*
Pro SP-C	Rabbit	Millipore	AB-3786	IF, ICC
Sca-1	Mouse	R&D	AF1226	ND*
Sox2	Mouse	Millipore	MAB4343	ND*
Sox2	Mouse	R&D	MAB2018	ND*
SSEA-1	Mouse	Imgenex	MC-480	ND*
TTF-1	Mouse	Thermo	MS-699	IF
Tubulin IV	Mouse	BioGenex	MU178-UC	IF, ICC, IHC

*: The detail information on the antigen, host, catalog number, vendor and applications of primary antibodies tested in this study was listed. ND: non-positive signal was detected in this study. CCSP, Clara cell secretory protein; ICC, immunocytochemistry; IF, immunofluorescence; IHC, immunohistochemistry; ITGA6, Integrin alpha 6; Sca-1, stem cells antigen-1; SSEA-1, stage-specific embryonic antigen-1; TTF-1, thyroid transcription factor 1.
